# Retention of Prey Genetic Material by the Kleptoplastidic Ciliate *Strombidium* cf. *basimorphum*

**DOI:** 10.3389/fmicb.2021.694508

**Published:** 2021-07-28

**Authors:** Maira Maselli, Konstantinos Anestis, Kerstin Klemm, Per Juel Hansen, Uwe John

**Affiliations:** ^1^Marine Biological Section, Department of Biology, University of Copenhagen, Helsingør, Denmark; ^2^Alfred-Wegener-Institute, Helmholtz Center for Polar and Marine Research, Bremerhaven, Germany; ^3^Helmholtz Institute for Functional Marine Biodiversity, Oldenburg, Germany

**Keywords:** kleptoplasty, ciliates, *Strombidium*, mixotrophy, plankton

## Abstract

Many marine ciliate species retain functional chloroplasts from their photosynthetic prey. In some species, the functionality of the acquired plastids is connected to the simultaneous retention of prey nuclei. To date, this has never been documented in plastidic *Strombidium* species. The functionality of the sequestered chloroplasts in *Strombidium* species is thought to be independent from any nuclear control and only maintained via frequent replacement of chloroplasts from newly ingested prey. Chloroplasts sequestered from the cryptophyte prey *Teleaulax amphioxeia* have been shown to keep their functionality for several days in the ciliate *Strombidium* cf. *basimorphum.* To investigate the potential retention of prey genetic material in this ciliate, we applied a molecular marker specific for this cryptophyte prey. Here, we demonstrate that the genetic material from prey nuclei, nucleomorphs, and ribosomes is detectable inside the ciliate for at least 5 days after prey ingestion. Moreover, single-cell transcriptomics revealed the presence of transcripts of prey nuclear origin in the ciliate after 4 days of prey starvation. These new findings might lead to the reconsideration of the mechanisms regulating chloroplasts retention in *Strombidium* ciliates. The development and application of molecular tools appear promising to improve our understanding on chloroplasts retention in planktonic protists.

## Introduction

Kleptoplasty is the non-permanent acquisition of chloroplasts from a photosynthetic organism by an otherwise heterotrophic organism (De Vries and Archibald, 2018). The phenomenon is common among marine ciliates (Stoecker et al., 1987; Stoecker et al., 2009). Since they acquire phototrophy from prey, plastidic ciliates are termed non-constitutive mixotrophs, or non-constitutive mixoplankton referring to planktonic species (Mitra et al., 2016; Flynn et al., 2019). Acquired phototrophy gives mixotrophic ciliates a competitive advantage over purely heterotrophic species when prey concentrations are low and light is available (Dolan and Pérez, 2000; Schoener and McManus, 2017).

Kleptoplastidic species in the *Mesodinium rubrum* species complex are known to only exploit chloroplasts from cryptophytes within the *Teleaulax/Plagioselmis/Geminigera* clade, from which they also retain the nuclei (process known as karyoklepty) and other prey organelles (Hansen et al., 2012; Johnson et al., 2016; Kim et al., 2017). The retention of prey nuclei allows the host to maintain some genetic control of the acquired chloroplasts through the transcription of plastid-related genes from the kleptokaryon. Other than the ability to photosynthesize, *Mesodinium rubrum* acquires from the prey the potential to metabolize several essential compounds including amino acids and vitamins (Altenburger et al., 2020). This enables *Mesodinium* species to retain fully functional plastids and live as a complete autotroph for about four generations in the absence of prey (Smith and Hansen, 2007). Such phenomena of kleptoplasty and karyoklepty have been also recorded in some dinoflagellates (Onuma and Horiguchi, 2015; Onuma et al., 2020).

Kleptoplastidic ciliates in the genera *Laboea*, *Strombidium*, and *Tontonia* can instead exploit chloroplasts derived from a much wider range of algal groups, including chlorophytes, haptophytes, cryptophytes, and heterokonts (Laval-Peuto and Febvre, 1986; Johnson and Beaudoin, 2019). These ciliates have much higher prey ingestion rates than *Mesodinium rubrum* and, thus, potentially a fast turnover of sequestered prey plastids. Photosynthesis contributes much less to the total carbon uptake compared to *M. rubrum*, and they cannot grow autotrophically when prey is not available (Schoener and McManus, 2012; Maselli et al., 2020). Transmission electron microscopy studies on kleptoplastidic ciliates in the genera *Laboea*, *Strombidium*, and *Tontonia* have never revealed the retention of any algal prey nuclei (Laval-Peuto and Febvre, 1986; Stoecker et al., 1988). The function of the sequestered chloroplasts in these ciliates is thus currently thought to depend on their innate robustness and ability to survive inside the ciliate host. Based on studies on the kleptoplastidic *Strombidium rassoulzadegani*, kleptoplastidic ciliates in the genus *Strombidium* are thought to depend on more frequent reacquisition of prey plastids compared to *M. rubrum* because they do not express genes related to plastid maintenance and replication (Santoferrara et al., 2014; Mcmanus et al., 2018).

*Strombidium basimorphum* is a worldwide distributed species, first morphologically described in Canadian waters (Martin and Montagnes, 1993) and then reinvestigated through molecular systematic in a Chinese population (Liu et al., 2011). It has been shown to significantly contribute to grazing on photosynthetic picoeukaryotes in a north Pacific ocean region (Orsi et al., 2018), but the retention of functional chloroplasts in this species has only recently experimentally ascertained on an isolate of *Strombidium* cf. *basimorphum* from Danish coastal waters (Maselli et al., 2020).

This ciliate seems to more efficiently exploit chloroplasts for photosynthesis when ingestion is suppressed by the unavailability of prey. Chlorophyll *a*-specific photosynthetic rates increase from about 2 pg C pg chl-a^–1^ day^–1^ when the ciliate actively ingest prey, to 6–8 pg C pg chl-a^–1^ day^–1^ when the prey gets depleted (Maselli et al., 2020; Hughes et al., 2021). Photosynthetic rates are kept relatively higher and constant during at least 5 days of prey starvation (Maselli et al., 2020). *Strombidium* cf. *basimorphum* can thus maintain chloroplasts functionality unaltered for several days when chloroplasts are not replaced via the ingestion of prey. To get some insight into the molecular mechanisms that stand behind the retention of functional chloroplasts, here, we tested the ability of the Danish isolate of *Strombidium* cf. *basimorphum* to also retain prey genetic material. We studied this in well-fed cells and in cells that had been starved for 1–7 days. Molecular techniques such as quantitative polymerase chain reaction (qPCR), fluorescence *in situ* hybridization (FISH) and single-cell transcriptomics were applied. Quantitative PCR and FISH, as applied in here on cultures, were recently developed to detect the presence of prey genetic material in *Mesodinium* cf. *major* in field samples (Herfort et al., 2017).

## Materials and Methods

### Culture Conditions and Experimental Design

Cultures of *S.* cf. *basimorphum* were established from single cells isolated from natural seawater samples from Roskilde fjord (Denmark). The isolate was identified and cultured as described in Maselli et al. (2020) and maintained for about 1 year feeding it the cryptophyte *Teleaulax amphioxeia* (SCCAP, K-1837). The experiment was conducted in *f*/20 media (a 1:10 dilution of the standard *f*/2 media from Guillard, 1975), at a salinity of 15, at 15°C, with a photon flux density of 100 μmol photons m^–2^ s^–1^ in a light/dark cycle of 14:10 h. Ciliates were allowed to grow exponentially for 5 days in borosilicate bottles (3.5 L of culture in 5-L flasks), by daily restoring the prey concentration that saturates their growth (*T. amphioxeia:* 1.0 × 10^4^ cell mL^–1^, as in Maselli et al., 2020). At the fifth day, ciliates were fed for the last time and split in three replicates of 1-L into 2-L Blue Cap glass flasks (VWR, Darmstadt, Germany). Cells were harvested for DNA extraction and FISH the day after, when prey was still available (T0); after 48 h, when prey was depleted (T2); and after 5 and 7 days, during prey starvation (T5 and T7).

### DNA Extraction

*S.* cf. *basimorphum* cells were collected from 200 mL of the experimental cultures onto Nitex nylon filters (Millipore by Merck, Darmstadt, Germany) with a mesh size of 10 μm, allowing the separation of the ciliates from prey (*T. amphioxeia* length, ∼5 μm). Filters were subsequently rinsed with clean culture media to make sure that no *T. amphioxeia* cells were retained. Samples of *T. amphioxeia* triplicate monocultures were collected on 0.2-μm polycarbonate filters (Whatman Nuclepore, Cytiva, Freiburg, Germany). DNA from both ciliates and prey samples was extracted using the NucleoSpin Soil DNA isolation kit (Macherey-Nagel, Düren, Germany), following the manufacturer’s instructions. Elution was performed using a small volume (35 μL) of the elution buffer provided by the kit. The concentration of the DNA was estimated using a NanoDrop spectrophotometer (ND-1,000 Peqlab, Erlangen, Germany).

### Quantitative PCR

*T. amphioxeia* nuclear 28S ribosomal RNA (rDNA) D2 Unique Sequence Element (USE) primers (TxD2 1F and TxD2 USE 2R) and nucleomorph 28S rDNA D2 USE primers (TxNm 1F and TxNm 1R) were used in qPCR assays to detect the presence of prey genetic material in DNA extracted from ciliates and provide a semiquantitative estimation of its concentration over time, following prey depletion and starvation. The primers (Supplementary Table 1) were designed and checked for their specificity by Herfort et al. (2017). All qPCR assays were run in technical triplicates on a StepOnePlus Real-Time PCR system (Applied Biosystems, Foster City, CA, United States). One nanogram of ciliate DNA was added to the following PCR mixture: 5 μL FAST SYBR Green Master Mix (Applied Biosystems by Thermo Fisher Scientific, Bremen, Germany), 0.125 μL of each primer (final concentration, 125 nM), and nuclease free water to a final volume of 10 μL.

Technical triplicate assays of each of the *T. amphioxeia* DNA replicate were run at the same DNA concentration as for the ciliate DNA. Quantitative PCR reactions were run as follows: 40 cycles of 95°C for 3 s and 60°C for 30 s, followed by a melting curve protocol (95°C for 15 s, 60°C for 1 min, and 0.3°C increments with a 15-s hold at each step). Control assays as a general control for extraneous nucleic acid contamination were also subjected to qPCR amplification with purified water in place of DNA.

### Fluorescence *in situ* Hybridization

To investigate the potential transcriptional activity of prey nuclear material in *S.* cf. *basimorphum*, ciliates were hybridized a FISH probe for the *T. amphioxeia* nuclear-encoded 28S rRNA D2 USE (TxD2 RNA, Table 1) designed by Herfort et al. (2017). Twenty milliliters of experimental cultures in the different growth phases (T0, T2, T5, and T7) were fixed in paraformaldehyde (4% final concentration) and stored at 4°C for 1 h, prior to the collection of the ciliates on 3-μm polycarbonate filters (Whatman Nuclepore). Filters were incubated for 1 h in 1 mL of 50% dimethylformamide (DMF) to reduce chloroplast autofluorescence (Groben and Medlin, 2005). Filters were subsequently hybridized for 3 h at 37°C in a buffer with 30% formamide according to Herfort et al. (2017), washed for 10 min at the same temperature with a second buffer [1 × SET buffer: 150 mM NaCl, 1 mM ethylenediaminetetraacetic acid (EDTA), 20 mM Tris/HCL], and counterstained with 4′,6-diamidino-2-phenylindole (DAPI). Samples were inspected using the Olympus BX50 microscope equipped with a CoolLED pE-300 light source on 400 × magnification with appropriate wavelengths for DAPI (excitation, 350 nm; emission, 450 nm), Alexa488 (excitation, 480 nm; emission, 530 nm), and chloroplast fluorescence (excitation, 600 nm; emission, 650 nm). Images were acquired by an Olympus DP71 camera using the software CellSense. *S.* cf. *basimorphum* samples from cultures fed with the green alga *Nephroselmis rotunda* were treated in the same way and used as negative control to check for the specificity of the probes.

**TABLE 1:** Average cycle threshold (Ct) values for prey nuclear and nucleomorph 28S genes in qPCR assays conducted on prey DNA (*T. amphioxeia* monoculture) and DNA extracted from the ciliate (*S.* cf. *basimorphum*) at different time points (corresponding to different nutritional stages of the ciliate culture).

	**Nuclear 28S rDNA**		**Nucleomorph 28S rDNA**	
	**No. of replicates**	**Average Ct**	**No. of replicates**	**Average Ct**
*T. amphioxeia* monoculture	9	20 ± 0.4	9	20.7 ± 0.6
T0, *S.* cf. *basimorphum*	9	29.2 ± 0.4	9	27.4 ± 0.4
T2, *S.* cf, *basimorphum*	9	34.1 ± 1.3	8	33.5 ± 1.7
T5, *S.* cf. *basimorphum*	7	35.5 ± 0.8	5	32.9 ± 1.4

*Ct values are reported as average ± standard deviation.*

### Single Cell Transcriptomics

To further validate the presence of prey transcripts in the ciliate as an indicator of active nuclei, nucleomorph, and/or plastid genome, single-cell transcriptomics was performed. Eight single cells were isolated from the experimental cultures after 4 days of prey starvation. Each cell was individually picked with a drawn Pasteur pipette, washed three times by transferring it in clean drops of sterile filtered media, and then transferred in the Lysis buffer provided by the extraction RNAqueous^TM^-Micro Total RNA Isolation Kit (Thermo Fisher Scientific, Bremen, Germany). The complementary DNA (cDNA) libraries were generated using the SMART-Seq v4 Ultra Low Input RNA Kit for Sequencing (Takara Bio Europe SAS, Saint-Germain-en-Laye, France), and cDNA was quantified using the Agilent High Sensitivity Kit (Agilent Technologies Germany GmbH & Co. KG, Waldbronn, Germany). Adapter and index ligation was done using the Nextera^®^ XT DNA Library Preparation Kit (Illumina GmbH, Berlin, Germany). The raw sequences were demultiplexed with bcl2fastq, and their quality and potential contamination with adapters were checked using FastQC. Reads were trimmed using TrimGalore with the default settings, and rRNA was removed with SortMeRNA (Kopylova et al., 2012). The final reads were mapped toward the reference transcriptome of *T. amphioxeia* generated by Altenburger et al. (2020), using the kallisto software (Bray et al., 2016). The reference assembly included the functional annotation of the transcripts with assigned Kyoto Encyclopedia of Genes and Genomes (KEGG) orthologs. To consider a *T. amphioxeia* transcript present in the ciliate, a threshold of ≥ 50 reads summed from all eight cells was set. The raw read sequences have been deposited at the National Center for Biotechnology Information (NCBI) under the BioProject PRJNA718746.

## Results

The *Strombidium* cf. *basimorphum* cultures decline in cell concentration immediately after prey was depleted. Ciliates concentration changed from ∼130 to 110 cells mL^–1^ during the two first days of the incubation to further decrease to ∼85 cells mL^–1^ after 3 days of prey starvation (T5) and to ∼60 cells mL^–1^ after 5 days of prey starvation (T7) (Supplementary Figure 1).

Despite the differences in harvested amount of cells at each time point, the yield of DNA extraction was similar ranging between 7.7 and 5.2 ng μL^–1^. However, 1 ng of template DNA extracted from ciliates that were actively feeding (T0) was sufficient to detect *T. amphioxeia* nuclear and nucleomorph 28S rDNA using the qPCR assays. The relative concentration of these prey genes appears to be lower in DNA extracted from ciliates subjected to prey deprivation, and only very low residual signals were detected after 3 days of starvation based on the average cycle threshold (Ct, Table 1). The amplification products of DNA extracted from ciliates at T7 were not reliable, so results of the qPCR assays for this time point are not shown. The reliability of the amplification products was assessed inspecting the melt curve of each of the replicate. Replicates that displayed multiple or shifted peaks in their melt curve have been omitted. The average cycle threshold (Ct) values for the nuclear 28S rDNA range between 29.2 ± 0.4 at T0 and 35.5 ± 0.8 at T5, while average Ct for the nucleomorph 28S rDNA is 27.4 ± 0.4 at T0 and 32.9 ± 1.4 at T5 (Table 1).

Average cycle thresholds (Cts) of the nuclear and nucleomorph genes are significantly different in *S.* cf. *basimorphum* at T0 (*p* < 0.0001), while they are not different in the *T. amphioxeia* monoculture.

**FIGURE 1:**
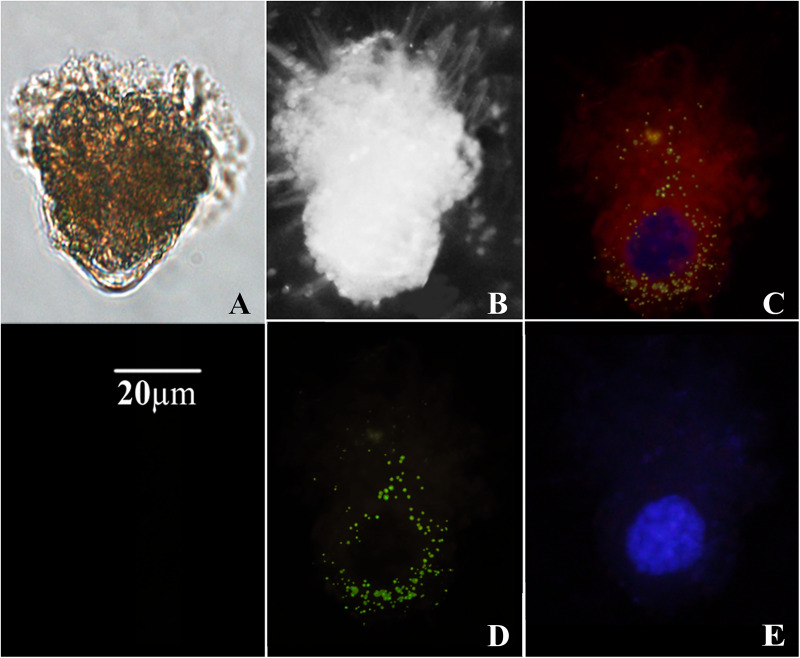
Micrographs of *Strombidium* cf. *basimorphum.* **(A)** Light microscopy of a *S.* cf. *basimorphum* cell in liquid suspension (prior the collection on filter). **(B)** Bright field micrograph of a *S.* cf. *basimorphum* cell on filter. **(C)** Micrograph of the same cell on filter, acquired with combined light channels: the cell is hybridized with the probe for the prey rRNA (green) and counterstained with DAPI (blue). Sequestered chloroplasts are visible in red. **(D)** Micrograph of the same cell acquired with a single light channel for the FISH probe, showing prey rRNA. **(E)** Micrograph of the same cell acquired with a single light channel for DAPI, showing *S.* cf. *basimorphum* macronucleus.

**FIGURE 2:**
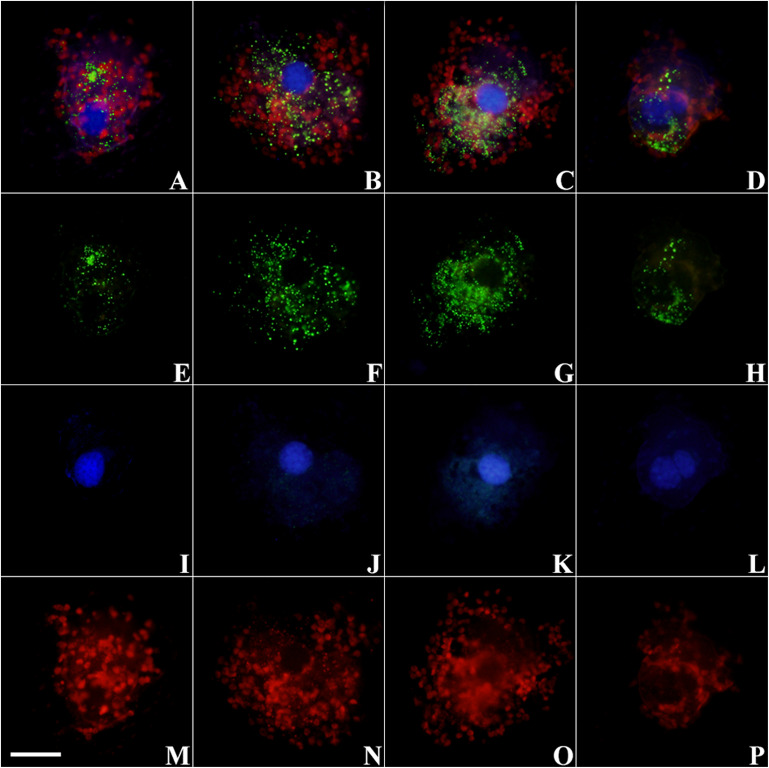
Micrographs of *S*. cf. *basimorphum* cells hybridized with the probe for the prey rRNA (Alexa Fluor 488 dye labeled) and counterstained with DAPI at different time intervals. Combined light channels: **(A)** T0, actively feeding cells; **(B)** T2, prey depleted; **(C)** T5, prey starved; **(D)** T7, prey starved. Single light channels are shown below each cell: **(E–H)** light channel for the FISH probe, showing prey rRNA; **(I–L)** light channel for DAPI, showing ciliates nuclei; and **(M–P)** light channel for chlorophyll autofluorescence, showing chloroplasts. The scale bar in the left bottom corner is 20 μm and refers to all panels. Blue, ciliates nuclei; green, prey RNA; red, chloroplasts.

The morphology of ciliates collected on filters was quite well preserved (Figure 1). The fluorescent signal obtained upon hybridization with *T. amphioxeia* rRNA probe was clearly detectable within the ciliate cytoplasm (Figures 1C,D, 2A–H).

Prey rRNA is quite spread inside the ciliate cells, but the fluorescent signal is more intense around the ciliate nuclei (Figures 1C,D, 2B,C) or in localized clusters within the ciliate cytoplasm (Figures 2A,E). The fluorescent signal of the prey rRNA probe could be detected in ciliates at all-time points (Figure 2), and its intensity seems comparable among individuals sampled at different time points (Figures 2E–H). Individual cells that contained labeled rRNA as wells as individual cells that did not were found in all samples. However, the percentage of positive hybridized cells (over total cell numbers) was not determined. Negative controls of *S*. cf. *basimorphum* fed with a different prey item (*N. rotunda*) did not show any signal of hybridization, confirming the specificity of the probes.

**FIGURE 3:**
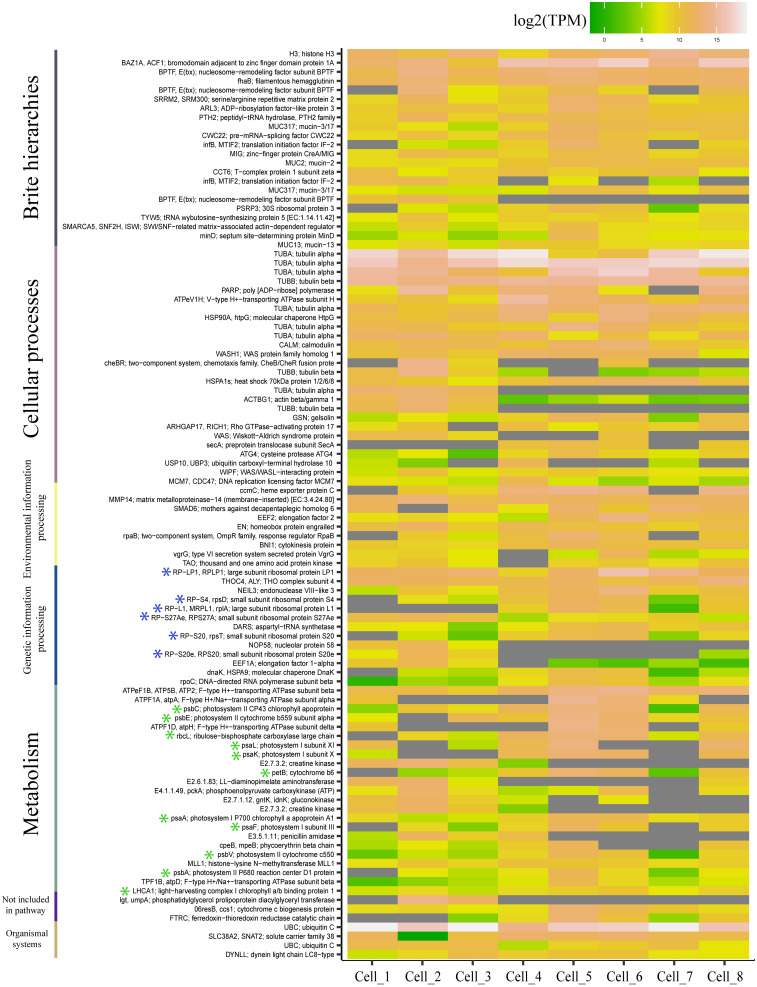
Heatmap representing the 100 most expressed transcripts of prey origin and their corresponding pathways according to the Kyoto Encyclopedia of Genes and Genomes. The expression values are provided for each separate cell and are shown as transcripts per million (TPM). Transcripts that have not been detected are represented in gray. Transcripts of ribosomal and chloroplast origin are highlighted with blue and green asterisks, respectively. The figure was generated using the R package ggplot2.

The transcriptomic analysis of the ciliate single cells revealed the presence of transcripts of prey nuclear origin. For each ciliate cell, an average of ∼17 million reads were generated and mapped against the reference transcriptome of *T. amphioxeia.* The mapping revealed the presence of 282 transcripts of prey nuclear and chloroplast origin (Supplementary Table 2). Among the 100 most expressed genes of prey origin, there were 11 transcripts of chloroplast origin and six transcripts encoding ribosomal proteins (Figure 3). Chloroplast genes included photosystems I and II apoproteins, subunits, and cytochromes. Moreover, we detected prey nuclear-encoded genes involved in amino acid biosynthesis and degradation. Genetic information pathways included genes related to the transcription and translation of the prey nucleus within the host. A detailed list of the retrieved transcripts and their functional annotation is provided in the Supplementary material.

## Discussion

The retention of prey genetic material is here documented for the first time in a kleptoplastidic *Strombidium* species through the use of specific molecular markers and single-cell transcriptomics. *Strombidium* cf. *basimorphum* is shown to retain genetic material from prey nuclei and nucleomorphs. The observation of prey rRNA and other transcripts of prey nuclear origin suggests that prey genetic material is transcriptionally active inside the ciliate.

Quantitative PCR results suggest that prey DNA disappears quite quickly after ingestion in *S.* cf. *basimorphum*, contrarily to what has been observed in *Mesodinium rubrum*, which is able to retain prey nuclei for up to 10 weeks (Johnson and Stoecker, 2005; Kim et al., 2017).

The relative concentration of the prey nucleomorph gene was higher compared to that of the prey nuclear gene (lower Ct values) in DNA-extracted *S.* cf. *basimorphum* but not in the DNA extracted from the prey monoculture, suggesting that prey nucleomorphs are better preserved in the ciliates compared to the prey nuclei. The reason for that may be attributed to the location of the nucleomorph in between the membranes of the chloroplasts of *T. amphioxeia* (Garcia-Cuetos et al., 2010). The location of the nucleomorph in between chloroplasts membranes would eventually preserve it from degradative processes in the ciliate cytoplasm. The presence of the nucleomorph could actually render *Teleaulax* chloroplasts favorable in comparison to other chloroplast types (Altenburger et al., 2020).

The strong fluorescent signal obtained upon hybridization with the prey rRNA probe proves the presence of prey ribosomes within the ciliate. It is not unequivocally proven that those ribosomes are being actively transcribed from the prey nuclear gene. Indeed, ribosomes could have been sequestered from the prey together with chloroplasts, although the general turnover rates make this not the most likely scenario. It is possible that rRNA clusters visualized with FISH in some of the ciliate cells are in fact food vacuoles. However, the fluorescent signal was persistent and diffuse all over the ciliate cytoplasm even after 5 days of prey starvation, suggesting that prey ribosomes are at least somehow maintained in the ciliate and are not only contained concentrated in food vacuoles. Nevertheless, a fraction of cells (not quantified) did not show any fluorescence upon hybridization. This can be ascribed to a dilution of the sequestered genetic material due to cell division, as has been described before in *Mesodinium rubrum* (Kim et al., 2017).

The transcriptional activity of the prey genetic material is proven by the results of the ciliate single-cell transcriptomics. The functional annotation of prey transcripts found in *S.* cf. *basimorphum* revealed the presence of genes of nuclear and chloroplast origin, involved in metabolic processes related to photosynthesis as well as to processes related to transcription and translation. All these processes argue for an active transcription of at least partially remained nuclei of the prey. These results will deserve further and extensive studies to elucidate the responses of the host toward functions related to the kleptoplasts (Uzuka et al., 2019) and the presence of photosynthesis-related genes (and eventually their evolutionary origin) within the genome of the host (Hongo et al., 2019; Hehenberger et al., 2019; Mansour and Anestis, 2021).

The fact that *S.* cf. *basimorphum*, unlike *Mesodinium*, is not able to grow as pure autotroph in the absence of prey could be explained by its need to incorporate nutrients other than carbon through ingestion. Further investigations of its transcriptome and the transcriptional activity of prey genetic material would provide further insight on the metabolism and potential dependence to prey metabolites of this ciliate.

Our study demonstrates the retention of prey genetic material in a *Strombidium* species; to which extent this is true for all plastidic *Strombidium* spp. is presently unknown. If retention of prey genetic material is indeed found in all plastidic *Strombidium* species, it would indicate that it is essential for the survival of plastids inside these ciliates. The ability (or lack of) to retain prey genetic material may also explain why kleptoplasts are not found in all *Strombidium* species and other ciliate groups living in the photic zone of the sea. To get deeper understandings, the same techniques would have to be employed on a natural specimen, and probes for different algal preys would have to be developed.

## Data Availability Statement

The datasets presented in this study can be found in online repositories. The names of the repository/repositories and accession number(s) can be found below: https://www.ncbi.nlm.nih.gov/, PRJNA718746.

## Author Contributions

MM, KA, KK, and UJ did the data acquisition and analysis. PH contributed to data interpretation and draft the manuscript. All authors contributed and commented on earlier versions of the manuscript and approved the final version of the manuscript, and involved in conceiving the study.

## Conflict of Interest

The authors declare that the research was conducted in the absence of any commercial or financial relationships that could be construed as a potential conflict of interest.

## Publisher’s Note

All claims expressed in this article are solely those of the authors and do not necessarily represent those of their affiliated organizations, or those of the publisher, the editors and the reviewers. Any product that may be evaluated in this article, or claim that may be made by its manufacturer, is not guaranteed or endorsed by the publisher.
