# Supplementary material for: Retention of Prey Genetic Material by the Kleptoplastidic Ciliate Strombidium cf. basimorphum
Source: Front Microbiol. 2021 Jul 28;12:694508. doi: 10.3389/fmicb.2021.694508 (PMC8355899; doi:10.3389/fmicb.2021.694508)
Supplement: Supplementary file 1 [file Data_Sheet_1.docx]

Supplementary Material

**Supplementary Table 1: primers and probes used in this study as designed by Herfort et al. 2017**

| **Assay** | **Gene region** | **Primer/probe** | **Sequence (5´–3´)** |
| --- | --- | --- | --- |
| qPCR | T. amphioxeianuclear 28S rDNAD2 | TxD2 1F | TGAAAAAGGGCCTGAAATTG |
|  |  | TxD2 2R | ATCATTCACTCGCATGCCCC |
|  | T. amphioxeianucleomorph28S rDNAD2 | TxNm 1F | ATCCTGGCGCGTGCTTAAAT |
|  |  | TxNm 1R | CTTCCGTCCGTCCTAAGAACA |
| FISH | T. amphioxeia nuclear-encoded 28S rRNA D2 | TxD2 rRNA | Alexa488AACACACGAGTTAAGATACCAATGGATCATTCACTCGCATGCCC |

**Table 2: Number of *T. amphioxeia* transcripts found in *S. cfbasimorphum*and their functional annotation according to KEGG**

| **KEGG pathway** | **Number of transcripts** |
| --- | --- |
| **Brite Hierarchies** | **94** |
| Protein families: genetic information processing | 72 |
| Protein families: metabolism | 1 |
| Protein families: signaling and cellular processes | 23 |
| **Cellular Processes** | **60** |
| Cell growth and death | 24 |
| Cell motility | 7 |
| Cellular community – eukaryotes | 7 |
| Cellular community – prokaryotes | 2 |
| Transport and catabolism | 15 |
| **Environmental Information Processing** | **21** |
| Membrane transport | 3 |
| Signal transduction | 18 |
| **Genetic Information Processing** | **44** |
| Folding, sorting and degradation | 10 |
| Replication and repair | 2 |
| Transcription | 9 |
| Translation | 23 |
| **Metabolism** | **50** |
| Amino acidmetabolism | 14 |
| Carbohydratemetabolism | 6 |
| Energy metabolism | 20 |
| Glycanbiosynthesis and metabolism | 1 |
| Lipidmetabolism | 2 |
| Metabolism of cofactors and vitamins | 2 |
| Metabolism of other amino acids | 2 |
| Nucleotidemetabolism | 1 |
| **Not Included in Pathway or Brite** | **5** |
| Unclassified: genetic information processing | 2 |
| Unclassified: metabolism | 3 |
| **Organismal Systems** | **11** |
| Development | 1 |
| Digestive system | 3 |
| Endocrine system | 5 |
| Excretory system | 2 |

**Supplementary Figure 1:** Changes in cell concentrations of *S.*cf*basimorphum* and *T. amphoxeia* in the experiment. Errors bars represent standard deviation among the three biological replicates.
